# Evaluation of larvicidal enhanced activity of sandalwood oil via nano-emulsion against *Culex pipiens* and *Ades aegypti*

**DOI:** 10.1016/j.sjbs.2022.103455

**Published:** 2022-09-23

**Authors:** Hanan Abo El-Kassem Bosly

**Affiliations:** Biology Department, Faculty of Science, Jazan University, Saudi Arabia

**Keywords:** Sandalwood oil, Nano-emulsion, Larvicidal, *Culex pipiens*, *Aedes aegypti*, Detoxifying enzymes

## Abstract

Mosquito control with essential oils is a trending strategy using aqueous oil nano-emulsions to expand their performance. Sandalwood essential oil and its prepared nano-emulsion used to estimate their larvicidal activities against the 3rd instar larvae of *Culex pipiens* and *Aedes aegypti* and their effects on larval tissue detoxifying enzymes. Sandalwood nano-emulsion was characterized by homogeneous, stable, average particles size (195.7 nm), polydispersity index (0.342), and zeta potential (−20.1 mV). Morphologically showed a regular spherical shape in size ranged from 112 to 169 nm that confirmed via scanning electron microscopy. Oil analysis identified sesquiterpene alcohols, mainly santalols, terpenoids, aromatic compounds, fatty acid methyl esters, and phenolic compounds. Larvicidal activities of the oil and its nano-emulsion indicated dose, formulation, and exposure time-related mortality after 24 and 48 h in both species. After 24 h, 100% mortality was detected at 1000 ppm for the nano-emulsion with LC_50_ of 187.23 and 232.18 ppm and at 1500 ppm for the essential oil with an LC_50_ of 299.47 and 349.59 ppm against the 3rd larvae *Cx. pipiens* and *Ae. aegypti*, respectively. Meanwhile, an enhanced significant effect of the nano-emulsion was observed compared to oil exposure in decreasing total protein content and the activities of alkaline phosphatase and β-esterase enzymes, and increasing α-esterase and glutathione S-transferase activities in larval body tissues. Results demonstrated the enhanced larvicidal potential of sandalwood oil nano-emulsion over that of oil. The effect involved alterations in the detoxifying enzymes based on the existing natural active ingredients against *Cx. pipiens* and *Ae. aegypti* larvae.

## Graphical abstract

**Figure:**
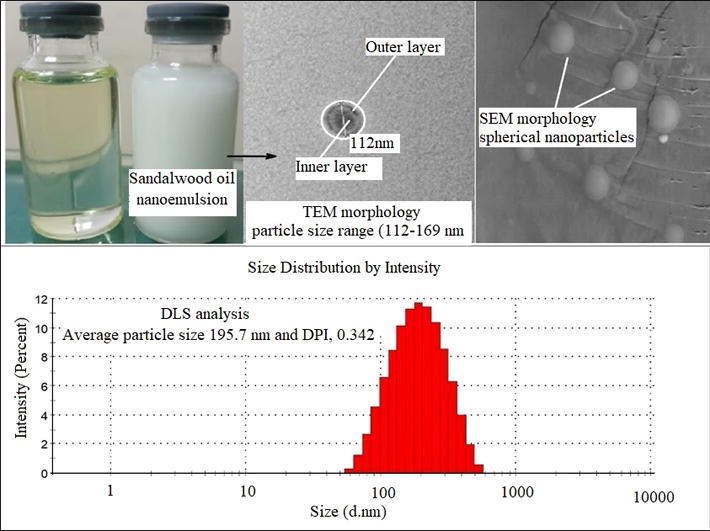

## 1 Introduction

Mosquitoes are important medical pests given their role in transmitting diseases among humans or animals. Vector control is the primary way to reduce public concerns about mosquito-borne diseases including filariasis, dengue fever, malaria, and leishmaniasis (Wilson et al., 2020). The control of larval stages of mosquito considered more efficient way to reduce the spread of mosquitoes than that of adults (WHO, 2013).

The search for environmentally friendly alternatives, like plants or oils, rich in secondary metabolites is a modern trend because of their efficiency, minimal toxicity, biodegradability, and the capability to reduce resistance (Şengül Demirak and Canpolat, 2022).

Nanotechnology is a multidisciplinary science that entails creating and using different systems and structures at the nanometer scale. Several forms of nano-emulsions, which are dispersed systems consisting of immiscible liquids and stabilizers, have been applied (McClements, 2012). Nano-emulsions are characterized by their thermodynamic stable and small droplets in size range 20–200 nm, leading to high efficacy (Jaiswal et al., 2015).

In Saudi Arabia, 51 mosquito species were recorded and the most abundant are *Ae. aegypti* and *Cx. pipiens* (Alahmed et al., 2019), with persisting insecticide resistance (Al-Sarar, 2010, Endersby-Harshman et al., 2021). In Saudi Arabia different studies of natural pesticides have been conducted against mosquito larvae, whether *Aedes aegypti* or *Culex pipiens*, due to the danger of disease transmission to humans especially in semi-desert areas, valleys, and other places (Al-Sarar, 2010, Bosly, 2015, Al-Massarani et al., 2019, El-Kasem Bosly, 2022).

Sandalwood oil, with scientific name, *Santalum album* L., Family: Santalaceae, a product of the wood and roots of sandalwood tree, is an essential oil widely found in India and East Asian countries, as well as in the northern coast of Australia and Hawaiian island. Sandalwood tree is expensive worldwide as its products are used all over the world due to its great economic importance. Sandalwood essential oil was identified to contain >150 terpenoid compounds, majority of which are α and β-santalol components, as well as others minor components including α-santalene, β-santalene, and α-bergamotene (Zhang et al., 2019). The oil, as well as the main compounds, have low toxicity upon oral and dermal exposure in experimental animals and showed antioxidant and anti-inflammatory effects, reflecting its protective activity in a cerebral ischemia mouse model (Younis and Mohamed, 2020).

This study was designed to evaluate the larvicidal efficacy of *Santalum album* oil and its nano-emulsion against *Culex pipiens,* and *Aedes aegypti* 3rd instar larvae, and determine their effect on the detoxifying enzymes activity in larval tissues. Oil constituents’ determination via gas chromatography-mass spectrometry analysis and oil phenolic compounds determination via liquid chromatography coupled with electrospray ionization and tandem mass spectroscopy.

## 2 Materials and methods

### 2.1 Chemicals

Tween 20, sodium glycocholate and sodium cholate hydrate were obtained from Alfa Aesar, Germany. Sandalwood oil purchased from the local market in jazan. Gallic acid, 3.4-dihydroxybenzoic acid, chlorogenic acid, catechin, caffeic acid, methyl gallate, syringic acid, coumaric acid, vanillin, rutin, ellagic acid, ferulic acid, myricetin, daidzein, luteolin, quercetin, naringenin, apegenin, kaempferol and hesperetin as phenolic compounds standards were purchased from Sigma Aldrich, USA. All reagents were HPLC grade.

### 2.2 Oil nano-emulsion preparation

The oil in water nano-emulsion was prepared by mixing 5 ml of sandalwood oil (at 45 °C) in 50 ml beaker contained 10 ml distilled water, 0.5 g sodium cholate, 0.5 g sodium glycocholate and 3.5 ml tween 20 (at 45 °C), stirred with magnetic stirrer until a clear emulsion was obtained. The mixture was quenched gradually with 50% v/v water, then, emulsified via sonication for 10 min at 200 W. The nano-emulsion was subjected to freeze drying lyophilization using SP Virtis Advantage Pro Laboratory Benchtop Freeze-Dryer Lyophilizer, with sucrose as a cryoprotectant (Yuan et al., 2008, Gundewadi et al., 2018).

### 2.3 Characterization of the oil nano-emulsion

#### 2.3.1 Particle size and surface charge using DLS and TEM analysis

The hydrodynamic radius and surface charge were investigated via dynamic light scattering (DLS) to determine the particle size (mean diameter) and zeta potential to confirm stability and uniformity by polydispersity index (PDI) and surface charge using zeta sizer nano Zs analyzer Malvern Panalytical, UK. One mg Sample was dispersed in 10 ml deionized water (Yuan et al., 2008). Particle morphology examined using TEM (Joel-1400 Flash) on carbon coated copper grids (600 mesh). Images were captured using CCD camera (EMT), the accelerating voltage was 80 kV (Yuan et al., 2008, Gundewadi et al., 2018).

#### 2.3.2 Scanning electron microscope (SEM)

Lyophilized sandalwood nano-emulsion sample used to obtain surface images via SEM (Quanta FDG 250, FEL, Hillsboro, OR, USA). The accelerating voltage 20 kV with 10.1 mm working distance (Dubes et al., 2003).

#### 2.3.3 Differential scanning calorimetry (DSC)

Lyophilized sandalwood nano-emulsion (5 g) was used to investigate the thermal stability profile (DSC-60, Shimadzu, Japan). The sample placed in standard aluminum pans with temperature raized from 2 to 200 °C covering the thermogram at 10 °C/min (Ji et al., 2016).

### 2.4 Gas chromatography-mass spectrometry (GC–MS)

Chemical composition of oil was determined using gas chromatography-mass spectrometry as detailed previously by El-Kasem Bosly (2022).

### 2.5 Liquid chromatography coupled with electrospray ionization and tandem mass spectroscopy (LC-ESI-MS/MS)

Phenolic compounds in the sandalwood oil sample were performed using LC-ESI-MS/MS for the separation. An ExionLC AC HPLC system and SCIEX Triple Quad 5500 + MS/MS system equipped with an electrospray ionization for detection. The column, ZORBAX SB-C18 (4.6 × 100 mm, 1.8 µm) was used. Two mobile phases, A: 0.1% formic acid in water and B: acetonitrile in programming mode as follows: 2% B from 0 to 1 min, 2 to 60% B from 1 to 21 min, 60% B from 21 to 25 min and 2% B from 25.01 to 28 min with 0.8 ml/min, as flow rate and the sample was 3 µl in volume. Positive and negative ionization modes were used in the same run in sittings for the multiple reactions monitoring (MRM) of the selected polyphenols as: curtain gas was 25 psi; for sitting positive and negative modes the IonSpray voltage were 4500 and-4500, respectively; source temperature was 400 °C; ion source gas 1 and 2 were 55 psi with a declustering potential at 50 V; collision energy at 25 eV and collision energy spread was 10 V.

### 2.6 *Culex pipiens* and *Aedes aegypti* mosquito colonies

Mosquito larvae of *Culex pipiens* and *Aedes aegypti* were reared as detailed by El-Kasem Bosly (2022).

### 2.7 Larvicidal assay

Larvicidal activities of sandalwood essential oil and its nano-emulsion were conducted against *Culex pipiens* and *Aedes aegypti* 3rd instar larvae according to WHO (2005). Two milliliters of the oil was placed in 100 ml water containing 2% tween 20 and subjected magnetic stirring (CR302, UK). Also, 2 ml of the prepared nano-emulsion was ultrasonicated in 100 ml water for equal distribution. Concentrations were prepared from the aforementioned preparations at 62.5, 125, 250, 500, 1000 and 1500 ppm. Twenty-five larvae from *Cx pipiens* and/or *Ae aegypti* were subjected to every-one concentration in glass beakers (250 ml in volume) comprising 150 ml of dechlorinated water (aqueous suspension) at 27 ± 2 °C, 70 ± 10% relative humidity and a 12:12 h light/dark photoperiod. The experiment was replicated five times for each concentration per extract and control group (solvent only treated). Larval mortalities were recorded after 24 and 48 h.

### 2.8 Larval preparation for biochemical assays

Third instar larvae of both species were exposed to oil and/or its nano-emulsion at the calculated LD_50_ in three replicates, as well as the control group, according to the aforementioned conditions in the larvicidal assay. Larvae were collected and weighed after 48 h from each group and pooled from each replicate for body homogenization in distilled water 10% (w/v) under ice and via cooling centrifugation at 4 °C for 15 min at 10000 rpm the supernatant was used for the biochemical assays.

### 2.9 Biochemical assays

Larvae supernatants were used for determination of total protein content (Bradford, 1976) and enzyme activities of alkaline phosphatase (Powell and Smith, 1954), α- and β-esterases (Van Asperen, 1962) and glutathione S-transferase (GST) (Habig et al, 1974).

### 2.10 Data analysis

Percentage larval mortality was calculated according to Abbott (1925). The larval control mortality was less than 5%, did not need correction according to the WHO, (2005) guidelines. Mortality and biochemical data resulting from all replicates were analyzed by one-way analysis of variance (ANOVA) to find the differences among the activity between each oil or nano-emulsion concentrations using the least significant difference test. Also, all replicates data were subjected to analysis for determination of the larval LC_50_, LC_90_, and LC_95_ as well as chi-square values within confidence limits at 95% by using probit analysis and regression between logarithm bas 10 of oil concentration and probit values. Data analysis was done via IBM SPSS Statistics v22 – 64 bit software with statistical significance at p < 0.05.

## 3 Results

### 3.1 Characteristics of oil nano-emulsion

**Fig. 1:**
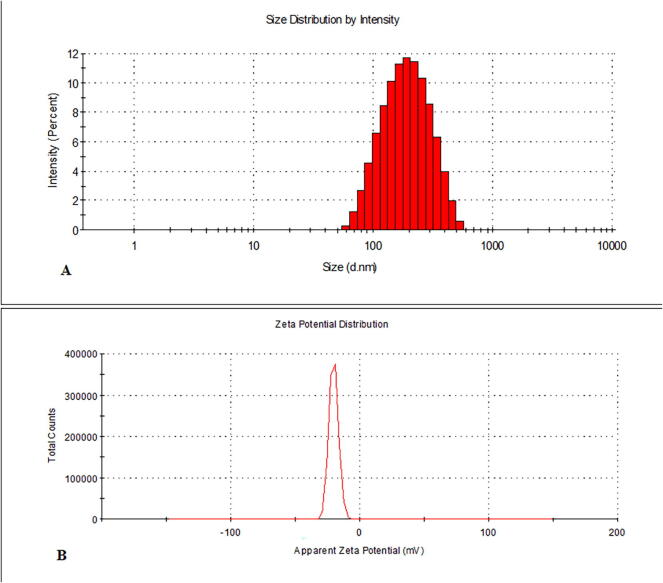
Dynamic light scattering and particle size analysis average particle size of 195.7 nm with PDI of 0.342 (A). Zeta potential and surface charge analysis (−20.1 mV) (B).

**Fig. 2:**
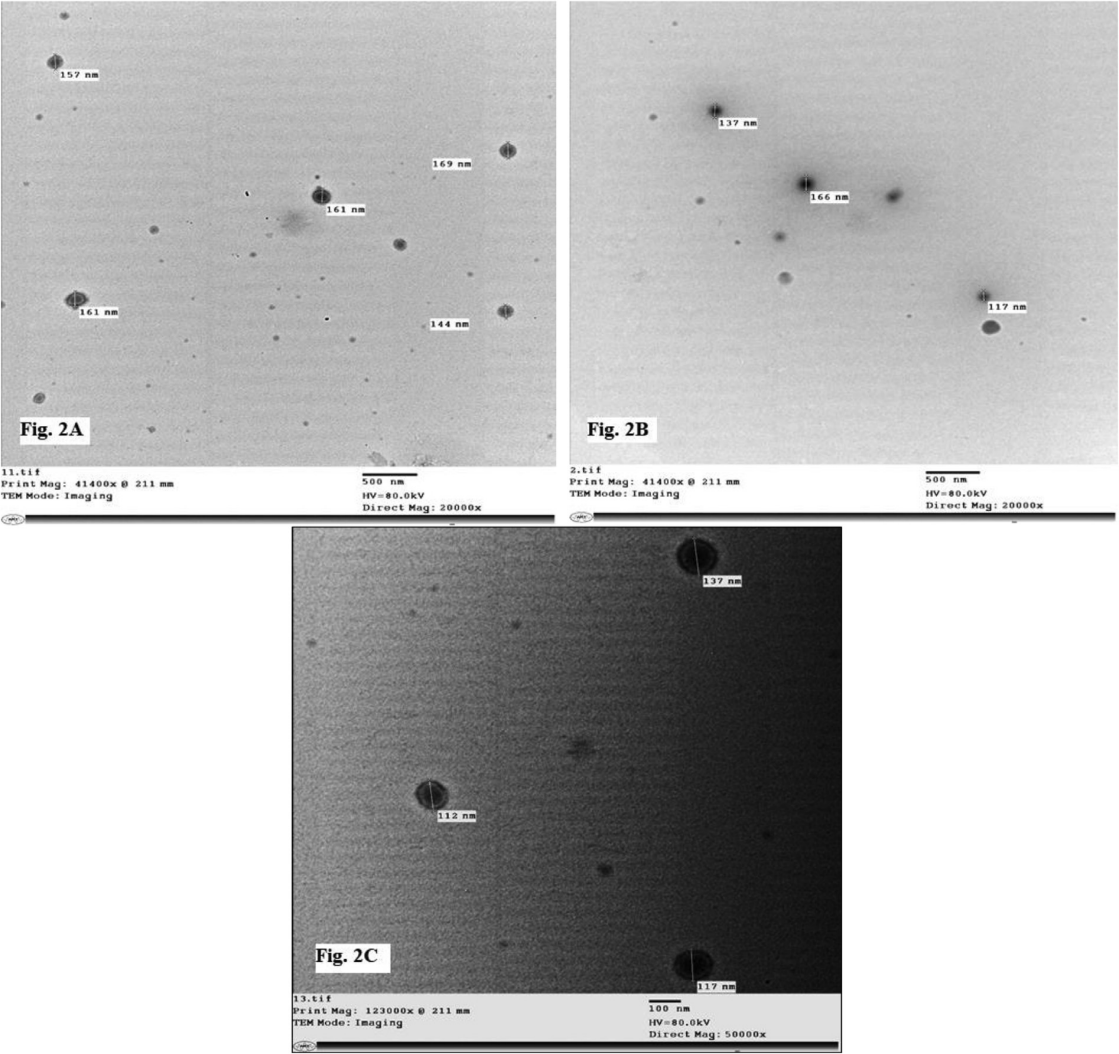
Transmission electron microscopy (TEM) of sandalwood oil nano-emulsion. Particle size (A) 144–169 nm, (B) 117, 137, and 166 nm, (C) 112–137 nm.

**Fig. 3:**
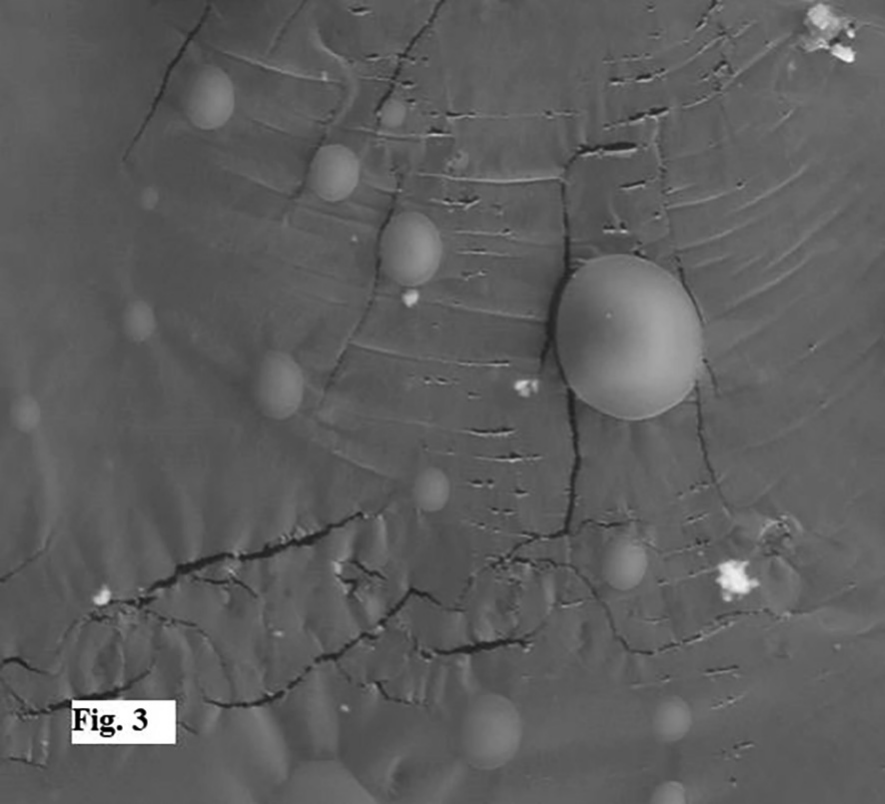
Scanning electron microscopy (SEM) of sandalwood oil nanoparticles (2000 × ).

**Fig. 4:**
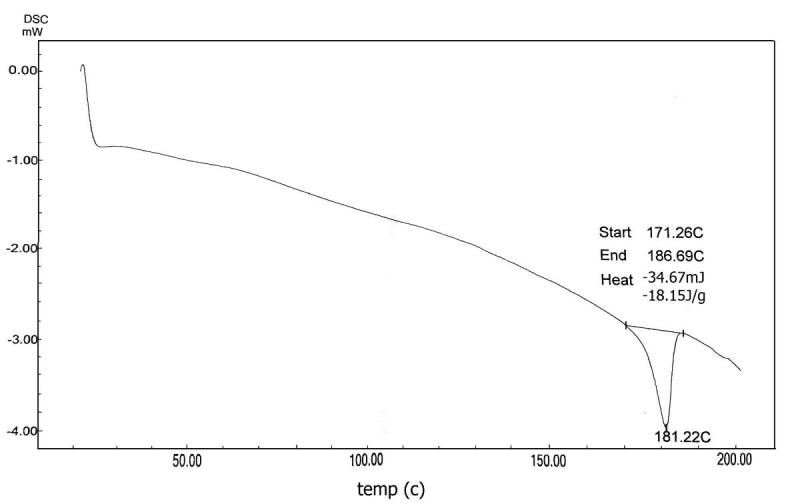
Differential scanning calorimetry of sandalwood oil nanoparticles.

Sandalwood oil nano-emulsion was characterized via DLS, which revealed particle average size of 195.7 nm and PDI of 0.342 (Fig. 1A), confirming homogeneous, stable and uniform narrow distributed nanoparticles. Zeta potential was −20.1 mV (Fig. 1B). The nano-emulsion morphology detected using TEM is represented in Fig. 2(A–C), showing regular spherical particles with a size in the range of 112–196 nm. Scanning electron micrograph (SEM) of lyophilized sandalwood nano-emulsion using sucrose as cryo-protectant was predicted smooth spherical particle shape (Fig. 3). Meanwhile, DSC thermogram showed an endothermic melting peak at 181 °C (Fig. 4).

### 3.2 Constituents of sandalwood oil by chromatographic analysis

**Fig. 5:**
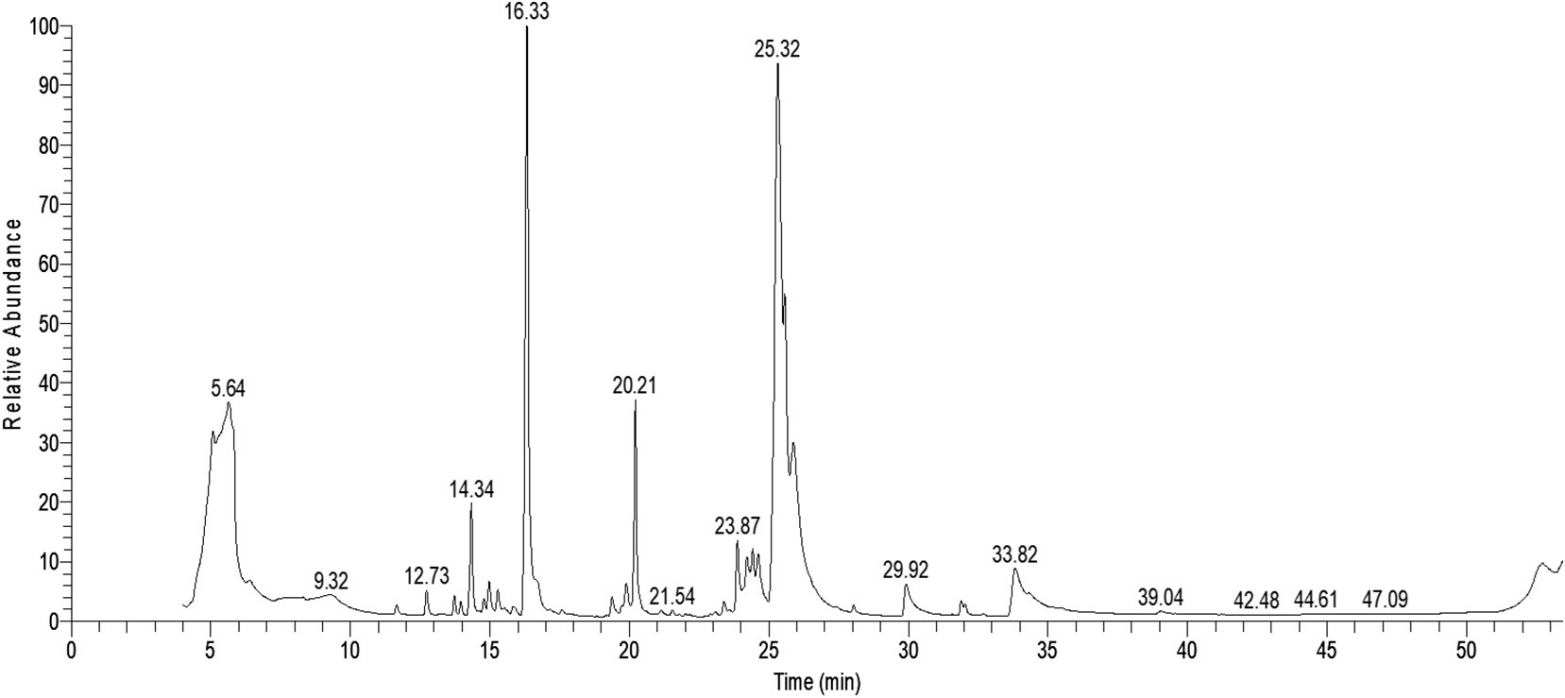
The total ion current chromatograms of sandalwood essential oil chemical constituents detected via GC–MS.

Gas chromatography-mass spectrometry analysis identified 27 compounds, sesquiterpene alcohols mainly santalols, terpenoids, aromatic compounds, and fatty acid methyl esters (Table 1 and Fig. 5). While phenolic compounds that were identified and quantified using LC-ESI-MS/MS analysis were chlorogenic acid (1.15426 ng/ml), ellagic acid (0.14722 ng/ml), luteolin (0.39093 ng/ml), and the highest content was recorded for naringenin (38.08662 ng/ml) (Table 2).

**Table 1:** Chemical constituents of sandalwood essential oil by gas chromatography-mass spectrometer (GC–MS).

No	Molecular formula	Chemical name	Area (%)	RT
1	C_6_H_12_O_3_	Butanoic acid, 2-hydroxy-, ethyl ester(Ethyl 2-hydroxybutanoate)	1.66	5.07
2	C_6_H_14_O_3_	1-Propanol, 2-(2-hydroxypropoxy)-(2-(2-Hydroxypropoxy)-1-propanol)	13.05	5.69
3	C_12_H_22_O_2_	Cyclohexanol, 4-(1,1-dimethylethyl)-(Cyclohexanol, 4-*tert*-butyl)	1.38	11.66
4	C_15_H_24_	α-Cedrene(1H-3a,7-Methanoazulene, 2,3,4,7,8,8a-hexahydro-3,6,8,8-tetramethyl-, [3R-(3α,3aβ,7β,8aα)]-)	0.78	13.73
5	C_15_H_24_	Caryophyllene(Bicyclo[7.2.0]undec-4-ene, 4,11,11-trimethyl-8-methylene-, [1R-(1R*,4E,9S*)]-)	0.52	13.96
6	C_13_H_20_O_2_	Nopyl acetate(2-Norpinene-2-ethanol, 6,6-dimethyl-, acetate)	4.33	14.34
7	C_12_H_20_O_2_	β Ionol(3-Buten-2-ol, 4-(2,6,6-trimethyl-1-cyclohexen-1-yl)-)	0.48	14.79
8	C_12_H_20_	1H-Indene, 1-ethylideneoctahydro-7a-methyl-, cis-((1Z)-1Ethylidene-7a-methyloctahydro-1H-indene)	2.09	14.97
9	C_15_H_24_	(+)-Sativene 1,4-Methano-1H-indene, octahydro-4-methyl-8-methylene-7-(1-methylethyl)-, [1S-(1α,3aβ,4α,7α,7aβ)]-	0.50	15.84
10	C_15_H_24_	Santalol, cis,α-2-Penten-1-ol,5-(2,3-dimethyltricyclo[2.2.1.02,6]hept-3-yl)-2-methyl-, (S)-(Z)-(−)- (8CI)	24.27	16.33
11	C_15_H_26_	Patchoulane 1H-3a,7-Methanoazulene, octahydro-1,4,9,9-tetramethyl-	0.53	16.72
12	C_15_H_26_O	Cedrol 1H-3a,7-Methanoazulen-6-ol, octahydro-3,6,8,8-tetramethyl-, [3R-(3α,3aβ,6α,7β,8aα)]-	0.73	19.37
13	C_14_H_26_O	Dodeca-1,6-dien-12-ol, 6,10-dimethyl-(6Z)-3,7-Dimethyl-6,11-dodecadien-1-ol	1.09	19.89
14	C_15_H_28_	4αH-Eudesmane Naphthalene, decahydro-1,4a-dimethyl-7-(1-methylethyl)-, [1S-(1α,4aα,7α,8aβ)]-	7.54	20.21
15	C_17_H_24_	Cycloisolongifolene, 8,9-dehydro-9-vinyl-	0.50	23.39
16	C_17_H_26_O	Acetyl cedrene 1H-3a,7-Methanoazulen-6-ol, octahydro-3,6,8,8-tetramethyl-, acetate, [3R-(3α,3aβ,6α,7β,8aα)]-	2.53	23.87
17	C_15_H_24_O	(Z) α-Santalol 2-Penten-1-ol, 5-(2,3-dimethyltricyclo[2.2.1.0(2,6)]hept-3-yl)-2-methyl-, [R(Z)]-	1.11	24.21
18	C_15_H_26_O	Isolongifolol 1,4-Methanoazulene-9-methanol, decahydro-4,8,8-trimethyl-, [1S-(1α,3aβ,4α,8aβ,9R*)]-	1.08	24.43
19	C_15_H_22_O	Longipinocarvone	1.32	24.62
20	C_12_H_20_O_2_	Tricyclodecandethanol Tricyclo(5.2.1.0(2,6))decanedimethanol	27.65	25.31
21	C_19_H_30_O_2_	13,16-Octadecadiynoic acid, methyl ester	0.30	28.04
22	C_18_H_34_O_2_	Oleic Acid	1.30	29.91
23	C_19_H_34_O_2_	9,12-Octadecadienoic acid, methyl ester, (E,E)-	0.62	31.89
24	C_19_H_36_O_2_	9-Octadecenoic acid (Z)-, methyl ester	0.44	32.03
25	C_18_H_32_O_2_	9,12-Octadecadienoic acid (Z,Z)-	2.31	32.80
26	C_20_H_36_O_2_	Linoleic acid ethyl ester	0.75	52.60
27	C_28_H_44_O_4_	9-Octadecenoic acid, (2-phenyl-1,3-dioxolan-4-yl)methyl ester, cis-	1.13	52.69

**Table 2:** Phenolic compounds standards (STD) versus a sample of sandalwood oil and their quantities (ng/ml) by LC-MS/MS.

No.	Compound	MRM Transition (m/z)	STD (80 ng/ml)		Sandalwood oil	Quantity	
			Area	RT	Area	RT	ng/ml
1	Gallic acid	168.9 > 124.9	446,000	3.83	ND	ND	ND
2	3.4-Dihydroxybenzoic acid	152.9 > 109	382,600	5.72	ND	ND	ND
3	Chlorogenic acid	355.1 > 163	668,900	7.31	6435	7.3	1.15426
4	Catechin	288.8 > 244.9	183,600	7.32	ND	ND	ND
5	Methyl gallate	183 > 124	6,739,000	7.42	ND	ND	ND
6	Caffeic acid	178 > 135	4,999,000	8.02	ND	ND	ND
7	Syringic acid	196.8 > 181.9	99,270	8.36	ND	ND	ND
8	Coumaric acid	162.9 > 119	7,477,000	9.48	ND	ND	ND
9	Vanillin	151 > 136	115,400	9.5	ND	ND	ND
10	Rutin	609 > 299.9	2,810,000	9.65	ND	ND	ND
11	Ellagic acid	301 > 145	47,300	9.86	5171	9.85	0.14722
12	Ferulic acid	192.8 > 133.9	299,700	10.18	ND	ND	ND
13	Myricetin	317 > 137	5011	11.64	ND	ND	ND
14	Daidzein	255.1 > 199	3,142,000	12.84	ND	ND	ND
15	Luteolin	284.7 > 132.9	3,174,000	13.42	15,510	13.43	0.39093
16	Querectin	301 > 151	2,015,000	13.49	ND	ND	ND
17	Cinnamic acid	146.9 > 102.6	44,290	14.09	ND	ND	ND
18	Naringenin	271 > 119	61,880	14.91	29,460	14.88	38.08662
19	Apigenin	269 > 151	23,740	14.95	ND	ND	ND
20	Kaempferol	284.7 > 93	416,600	15.24	ND	ND	ND
21	Hesperetin	301 > 136	1,007,000	15.52	ND	ND	ND

ND, not detected; MRM, multiple reactions monitoring; RT, retention time; STD, standard.

### 3.3 Larvicidal activities

Larval mortality data are represented in Table 3. In sandalwood oil -exposed groups, after 24 h, *Cx. pipiens* and *Ae aegypti* larvae exhibited 100% mortality at a dose of 1500 ppm with LD_50,_ LD_90_ and LD_95_ identified as 299.47, 847.81, and 1138.73 ppm for *Cx. pipiens* and 349.59, 1011.54, and 1367.06 ppm for *Ae aegypti*, respectively. After 48 h, 100% mortality observed at 1000 and 1500 ppm for *Cx. pipiens* and at 1500 ppm for *Ae aegypti* with LD_50,_ LD_90_, and LD_95_ identified as 213.01, 617.64, and 835.22 ppm for *Cx. pipiens,* and 250.64, 709.06, and 952.17 ppm for *Ae aegypti*, respectively.

**Table 3:** The larvicidal activities of sandalwood oil and sandalwood nanoemulsion against *Culex pipiens* and *Aedes aegypti* 3rd instar larvae post 24 and 48 h of exposure.

Oil type	Concentration (ppm)	Mortality% (Mean ± SEM)			
		*Culex pipiens*			*Aedes aegypti*
		24 h	48 h	24 h	48 h
Sandalwood oil	0.0	0.00 ± 0.00^a^	1.60 ± 0.98^a^	0.00 ± 0.00^a^	2.40 ± 0.98^a^
	62.5	4.80 ± 0.80^b^	11.20 ± 1.50^b^	4.00 ± 1.26^a^	8.00 ± 1.26^b^
	125	14.40 ± 0.98^c^	24.00 ± 4.56^c^	11.20 ± 1.96^b^	17.60 ± 2.71^c^
	250	38.40 ± 2.04^d^	52.80 ± 1.50^d^	32.00 ± 1.79^c^	46.40 ± 0.98^d^
	500	68.00 ± 1.79^e^	80.80 ± 3.20^e^	60.00 ± 1.26^d^	75.20 ± 2.33^e^
	1000	94.40 ± 1.60^f^	100.00 ± 0.00^f^	89.60 ± 2.71^e^	98.40 ± 1.60^f^
	1500	100.00 ± 0.00 ^g^	100.00 ± 0.00^f^	100.00 ± 0.00^f^	100.00 ± 0.00^f^
	LC_50_ (LCL-UCL)	299.47 (268–334)	213.01 (190–238)	349.59 (313–390)	250.64 (224–280)
	LC_90_ (LCL-UCL)	847.81 (727–1022)	617.64 (528–749)	1011.54 (864–1225)	709.06 (608–855)
	LC_95_ (LCL-UCL)	1138.73 (952–1422)	835.22 (695–1052)	1367.06 (1137–1717)	952.17 (795–1191)
	Chi^2^ (Sig)	6.340(0.18^a^)	9.308(0.54^a^)	8.783(0.67^a^)	8.813(0.66^a^)
	Reg. Eq.	Y = −6.6 + 2.67*x	Y = −5.5 + 2.34*x	Y = −6.35 + 2.49*x	Y = −6.86 + 2.89*x
	R^2^	0.987	0.990	0.989	0.963

Sandalwood nanoemulsion	0.0	0.00 ± 0.00^a^	1.60 ± 0.98^a^	0.00 ± 0.00^a^	2.40 ± 0.98^a^
	62.5	14.40 ± 1.60^b*^	19.20 ± 2.33^b*^	8.00 ± 1.26^b^	16.80 ± 1.50^b*^
	125	26.40 ± 2.04^c*^	39.20 ± 2.33^c*^	21.60 ± 0.98^c*^	32.80 ± 2.94^c*^
	250	57.60 ± 2.04^d*^	75.20 ± 3.67^d*^	48.00 ± 1.26^d*^	63.20 ± 3.20^d*^
	500	88.80 ± 1.96^e*^	98.40 ± 1.60^e*^	79.20 ± 3.20^e*^	92.80 ± 2.94^e*^
	1000	100.00 ± 0.00^f*^	100.00 ± 0.00^f^	100.00 ± 0.00^f*^	100.00 ± 0.00^f^
	1500	100.00 ± 0.00^f^	100.00 ± 0.00^f^	100.00 ± 0.00^f^	100.00 ± 0.00^f^
	LC_50_ (LCL-UCL)	187.23 (167–209)	137.44 (122.61–153.18)	232.18 (208.00–258.75)	182.37 (162.67–204.32)
	LC_90_ (LCL-UCL)	532.08 (455–645)	355.91 (306.02–430.98)	638.98 (548.92–769.41)	468.47 (400.33–569.57)
	LC_95_ (LCL-UCL)	715.42 (595–902)	466.10 (390–588)	851.39 (713–1063)	630.18 (523–798)
	Chi^2^ (Sig)	8.973(0.062^a^)	6.058(0.195^a^)	9.044(0.062^a^)	7.022(0.135^a^)
	Reg. Eq.	Y = −5.79 + 2.54*x	Y = −7.05 + 3.32*x	Y = −5.87 + 2.46*x	Y = −5.91 + 2.67*x
	R^2^	0.970	0.964	0.995	0.972

Significance at 0.05 level between different superscripts within the same column of each treatment. SEM, standard error of the mean; LCL, lower confidence limit; UCL, upper confidence limit. (*) reflects significance within the same concentration level between the two treatments within the same column.

In nano-emulsion-exposed groups, after 24 h, *Cx. pipiens* and *Ae aegypti* larvae exhibited 100% mortality at 1000 ppm, with LD_50,_ LD_90_, and LD_95_ identified as 187.23, 532.08, and 715.42 ppm for *Cx. pipiens,* and 232.18, 638.98, and 851.39 ppm for *Ae aegypti*, respectively. After 48 h 100% larval mortality was observed at 1000 and 1500 ppm with LD_50,_ LD_90_, and LD_95_ identified as 137.44, 355.91, and 466.10 ppm for *Cx. pipiens* and 182.37, 468.47, and 630.18 ppm for *Ae aegypti*, respectively.

### 3.4 Biochemical results

Biochemical results represented in Table 4, showing significant decrease in TP content and ALP and β esterase enzymes activities in *Cx. pipiens* and *Ae. aegypti* exposed to both treatments with significant lowering effect of nano-emulsion as compared to oil. Meanwhile, α esterase and GST enzymes activities showed significant increase upon both treatments as compared to corresponding controls. In addition, nano-emulsion exposed groups showed significant increase in α esterase and GST enzymes activities in *Cx. pipiens* and *Ae. aegypti* groups comparing to the parallel values in oil exposed groups.

**Table 4:** Effect sandalwood oil and sandalwood nanoemulsion at LD_50_ dose on *Culex pipiens* and *Aedes aegypti* 3rd instar larvae.

Parameter	Groups	Sandalwood oil	% change from control	Sandalwood nanoemulsion	% change from control
TP mg/g	Control	49.67 ± 1.02^a^		48.33 ± 0.33^a^	
	*Culex pipiens*	45.67 ± 0.67^*a^	−8.05	42.33 ± 1.20^*b^	−12.41
	*Aedes aegypti*	44.67 ± 0.67^*a^	−10.07	44.00 ± 0.00^*a^	−8.96

ALP mU/g	Control	3765.67 ± 57.25^a^		3762.00 ± 3.00^a^	
	*Culex pipiens*	1626.33 ± 18.89^*a^	−56.81	1509.33 ± 0.67^*b^	−59.88
	*Aedes aegypti*	1598.67 ± 30.78^*a^	−57.55	1466.67 ± 3.33^*b^	−61.01

α esterase μg α-naphthol/min/g	Control	667.67 ± 8.65^a^		681.00 ± 1.09^a^	
	*Culex pipiens*	766.67 ± 8.11^*a^	14.83	820.00 ± 12.17^*b^	20.41
	*Aedes aegypti*	747.33 ± 9.40^*a^	11.93	780.67 ± 5.33^*b^	14.64

β esterase μg β-naphthol/min/g	Control	464.33 ± 3.48 ^a^		455.00 ± 2.89^a^	
	*Culex pipiens*	321.67 ± 1.67^*a^	−30.72	298.00 ± 3.61^*b^	−34.51
	*Aedes aegypti*	323.00 ± 2.52^*a^	−30.44	313.67 ± 1.20^*b^	−31.06

GST m mole sub.Conjugated/min/g	Control	132.00 ± 2.31^a^		128.00 ± 2.31^a^	
	*Culex pipiens*	151.00 ± 2.08^*a^	14.39	185.33 ± 3.71^*b^	44.79
	*Aedes aegypti*	142.00 ± 1.15^*a^	7.58	161.33 ± 1.86^*b^	26.04

Data expressed as means ± SEM. SEM, standard error of the mean. Significance (p > 0.05) between larval groups represented by (*) superscripts as compared to their corresponding control within the same column. Different subscripts indicated significance between treatments (between columns). TP, total protein; ALP, alkalinphosphatase; GST, glutathione S-transferase.

## 4 Discussion

In recent years, there has been a great interest from health authorities and organizations in the significance of vector-borne diseases at the global and regional levels since they continue to demonstrate a significant health threatening to the societies worldwide (World Health Organization, 2017, Valenzuela et al., 2018). Mosquito-borne diseases represent the largest measure of this fear, that’s because of the mosquitoes ability to transmit many medical and veterinary diseases, like, filariasis, malaria, dengue fever, Rift Valley fever, Lumpy skin, and others which negatively affects human health and causes clear economic losses (Al-Seghayer et al., 1999, Singh et al., 2019). In addition to this interest, research on mosquito control based natural alternative agents instead of synthetic pesticides, with a clear appreciation in the scientific and medical community, especially natural products derived from plants (Şengül Demirak and Canpolat, 2022). Because of their capability to win the goal for reducing pests without harming the environment, essential oils within their chemical constituents, exerted beneficial effects and due to their lipophilic nature acquired the capability for crossing membranes and hence, exerts their toxicity activity towards insects, as well as their antimicrobial, antibacterial, antifungal, antiviral in line with their miscellaneous activities (Stephane and Jules, 2020).

The sandalwood oil nano-emulsion prepared in the present study characterized by Zeta potential was within range −30–30 mV associated with stable nano-emulsion systems and the negative value is necessary for droplet–droplet repulsion and enhanced nano-emulsion stability. In addition, the recorded small PDI that described the degree of particles distribution uniformity in the emulsion confirmed good homogeneity indication (Danaei et al., 2018, Gul et al., 2022). TEM findings agree with DLS data, however, the particle size determined using TEM was smaller than that detected using DLS due to the sensitivity of technique (Klang et al., 2012). The characteristics of the nano-emulsion were in agreement with previous studies (González et al., 2016, Firooziyan et al., 2021, Zamaniahari et al., 2022). Differential scanning calorimetry can be applied for the recognition of microsponges when loaded molecules are entrapped nearby. Melting, boiling, and/or sublimation points of the entrapped molecules generally change or disappear. According to this, the presented melting peak was thought to be the effect of the sucrose cryo-protectant and no significant endothermic peak was observed for the sandalwood oil nano-emulsion that it was liquid at room temperature.

Both GC–MS and LC-ESI-MS/MS analysis of sandalwood essential oil confirmed the constituents previously recorded (Butaud et al., 2006, Misra and Dey, 2012, Bisht et al., 2019, Kucharska et al., 2021, Tripathi et al., 2022).

The larval mortality results confirmed the sandalwood oil effect previously identified against *Ae. aegypti* larvae (Amer and Mehlhorn, 2006). In addition to the efficient larvicidal predicted action against *Cx pipiens, Ae aegypti* and *Aedes albopictus* larvae that reportedly due to the toxicity of the oil constituents (Zhu et al., 2008). Another study showed significant repellant and insecticidal activities of sandalwood oil and its main active ingredients α- and β- santalols against *Aphis gossypii* and suggested sandalwood oil and its main compounds for use as possible ecofriendly management against *Aphis gossypii* (Roh et al., 2015). Sandalwood oil showed a repellent activity for the parasitic mite, *Varroa jacobsoni* which invades and threatens honeybee colonies (Imdorf et al., 1999) and against *Lycoriella mali*, Sciarid flies, with modest activity reported (Choi et al., 2006). Besides, santalol showed activity against the spider mite *Tetranychus urticae* (Roh et al., 2012) acting as acaricidal and oviposition deterring. Furthermore, Indian sandalwood tree (*S. Album* L.) has benificial properties in inhibiting insects’ growth due to its chemical properties (Shankaranarayana et al. 1980).

The study results, showed a significant larvicidal efficacy of the nano-emulsion as compared to that of the oil against both larvae, revealing the enhanced activity of the nano-emulsion in agreement with Duarte et al. (2015), who evaluated rosemary essential oil nano-emulsion and its potential larvicidal effect against *Ae. aegypti* larvae. Moreover, Mahran (2022) evidenced the larvicidal improvement of basil and cumin essential oils in their nano-emulsion formulations against *Cx. pipiens* larvae.

The sandalwood oil nanoemulsion also recorded significant decrement in total protein contents in the exposed species as compared to their concentrations in the oil exposed larvae and both treatments showed total protein significant decrement as compared to control value, which proposed for the synthesis microsomal detoxifying enzymes (Massoud et al., 2001). The total protein decrement was confirmed in previous studies (Koodalingam et al., 2012, Sugumar et al., 2014). Esterases and GST function as detoxification enzymes for endogenous and exogenous chemicals to eliminate or transform them to less toxic metabolites through different metabolic pathways. The alteration of enzymes throughout the oil compounds action besides the role of enzymes in metabolizing oil constituents was previously proposed (Intirach et al., 2019). Sandalwood oil larvicidal activity was proposed through its target for the detoxifying enzymes (Tong and Bloomquist, 2013) which increased larval sensitivity to tannins and generally for phenolic compounds, in accordance with the predicted sandalwood oil compounds with proposed mosquitocidal activity (Rey et al., 1999, Rey et al., 2001).

Sandalwood oil showed antiviral activity against herpes simplex virus type 1 (HSV-1) in a dose dependent manner and the activity was proposed via oil increment effect on cellular GST enzyme activity (Benencia and Courrèges, 1999). Noting that β-esterase activity decreased in the present results, which is often the reverse of α-esterase activity as a saver for the larvae from the oil constituents’ toxicity. Also, could be because esterase proteins have different substrate specificities resulting in different active sites of the two esterases (Montella et al., 2012). The present results may support the involvement of that enzymes in the detoxification of sandalwood oil or its nanoemulsion in the tested larvae.

Essential oil nano-emulsion protects the oil against oxidation and controls its release and bioactivity by increasing the exposed area and providing the interaction of oil active compounds with their target, resulting in increased stability and shelf life, decreasing degradation due to environmental factors. These properties indicate their effectiveness compared to crude and even pure oil (da Silva et al., 2022). In previous study neem oil nano-emulsion showed effective larvicidal potency aginst *Cx. quinquefasciatus* 3rd instar larvae (Anjali et al., 2012). Balasubramani et al, (2017) showed the larvicidal activity advantage of *Vitex negundo* L. leaf essential oil nano-emulsion (particle size, 200 nm) against *Ae. aegypti* larvae as compared to that of the oil after 12 and 24 h. Firooziyan et al, (2021) reported increased larvicidal efficacy of *Cinnamim zelanicum* nano-emulsion against *An. stephensi* larvae compared to the essential oil. Similarly, *Aeollanthus suaveolens* Mart. leaves oil in the nano-emulsion formulation (particle size 126.73 nm and zeta potential −16.25 mV) evaluated larval toxicity *Ae. aegypti* larvae (Lopes Martins et al., 2021).

## 5 Conclusion

The study verified the enhanced larvicidal potential of sandalwood oil nano-emulsion against *Cx. pipiens* and *Ae. aegypti* mosquito larvae as compared to that of the oil as well as alterations in the detoxifying enzymes based on oil active ingredients. Although the rational use of sandalwood oil is limited as insecticide due to the coast, it is used in a wide range of applications in fragrance and medicinal usage. The insecticidal activity offers a variety of use as a pesticide and the nano-emulsion formulation adds extra stability and elevates its toxicity against the tested mosquito larvae. The study recommends sandalwood oil nano-emulsion as a safe and stable larvicide against *Cx. pipiens* and *Ae. aegypti* and more biochemical investigations are warranted to explore more larvicidal mode of action.

## Declaration of Competing Interest

The authors declare that they have no known competing financial interests or personal relationships that could have appeared to influence the work reported in this paper.
