# Supplementary material for: Evaluation of larvicidal enhanced activity of sandalwood oil via nano-emulsion against Culex pipiens and Ades aegypti
Source: Saudi J Biol Sci. 2022 Sep 23;29(12):103455. doi: 10.1016/j.sjbs.2022.103455 (PMC9523098; doi:10.1016/j.sjbs.2022.103455)
Supplement: Supplementary data 1 [file mmc1.pdf]

Evaluation of Larvicidal Enhanced Activity of Sandalwood Oil via Nano-emulsion against Culex Pipiens and Ades Aegypti

By Hanan Bosly

WORD COUNT	5497	TIME SUBMITTED	04-AUG-2022 11:53AM
		PAPER ID	89216682

Evaluation of Larvicidal Enhanced Activity of Sandalwood Oil via Nano-emulsion against *Culex Pipiens* and *Ades Aegypti*

ABSTRACT

Mosquito control with essential oils is a trending strategy using aqueous oil nano-emulsions to expand their performance. Sandalwood essential oil and its prepared nano-emulsion used to estimate their larvicidal activities against the 3rd instar larvae of *Culex pipiens* and *Aedes aegypti* and their effects on larval tissue detoxifying enzymes. Sandalwood nano-emulsion was characterized by homogeneous, stable, average particles size (195.7 nm), polydispersity index (0.342), and zeta potential (-20.1 mV). Morphologically showed a regular spherical shape in size ranged from 112 to 169 nm that confirmed via scanning electron microscopy. Oil analysis identified sesquiterpene alcohols, mainly santalols, terpenoids, aromatic compounds, fatty acid methyl esters, and phenolic compounds. Larvicidal activities of the oil and its nano-emulsion indicated dose, formulation, and exposure time-related mortality after 24 and 48 h in both species. After 24 h, 100% mortality was detected at 1000 ppm for the nano-emulsion with LC_{50} of 187.23 and 232.18 ppm and at 1500 ppm for the essential oil with an LC_{50} of 299.47 and 349.59 ppm against the 3rd larvae *Cx. pipiens* and *Ae. aegypti*, respectively. Meanwhile, an enhanced significant effect of the nano-emulsion was observed compared to oil exposure in decreasing total protein content and the activities of alkaline phosphatase and β-esterase enzymes, and increasing α–esterase and glutathione S-transferase activities in larval body tissues. Results demonstrated the enhanced larvicidal potential of sandalwood oil nano-emulsion over that of oil. The effect involved alterations in the detoxifying enzymes based on the existing natural active ingredients against *Cx. pipiens* and *Ae. aegypti* larvae.

Keywords: Sandalwood oil; Nano-emulsion; Larvicidal; *Culex pipiens; Aedes aegypti;* Detoxifying enzymes

1. Introduction

Mosquitoes are important medical pests given their role in transmitting diseases among humans or animals. Vector control is the primary way to reduce public concerns about mosquito-borne diseases including filariasis, dengue fever, malaria, and leishmaniasis (Wilson et al., 2020). The control of larval stages of mosquito considered more efficient way to reduce the spread of mosquitoes than that of adults (WHO, 2013).

The search for environmentally friendly alternatives, like plants or oils, rich in secondary metabolites is a modern trend because of their efficiency, minimal toxicity, biodegradability, and the capability to reduce resistance (Şengül Demirak and Canpolat, 2022).

Nanotechnology is a multidisciplinary science that entails creating and using different systems and structures at the nanometer scale. Several forms of nano-emulsions, which are dispersed systems consisting of immiscible liquids and stabilizers, have been applied (McClements, 2012). Nano-emulsions are characterized by their thermodynamic stable and small droplets in size range 20 - 200 nm, leading to high efficacy (Jaiswal et al., 2015).

In Saudi Arabia, 51 mosquito species were recorded and the most abundant are *Ae. aegypti* and *Cx. pipiens* (Alahmed et al., 2019), with persisting insecticide resistance (Al-Sarar, 2010; Endersby-Harshman et al., 2021). In Saudi Arabia different studies of natural pesticides have been conducted against mosquito larvae, whether *Aedes aegypti* or *Culex pipiens*, due to the danger of disease transmission to humans especially in semi-desert areas, valleys, and other places (Al-Sarar, 2010; Bosly, 2015; Al-Massarani et al., 2019; El-Kasem Bosly, 2022).

Sandalwood oil, with scientific name, *Santalum album* L., Family: Santalaceae, a product of the wood and roots of sandalwood tree, is an essential oil widely found in India and East Asian countries, as well as in the northern coast of Australia and Hawaiian island. Sandalwood tree is expensive worldwide as its products are used all over the world due to its great economic importance. Sandalwood essential oil was identified to contain greater than 150 terpenoid compounds, majority of which are α and β-santalol components, as well as others minor components including α-santalene, β-santalene, and α-bergamotene (Zhang et al., 2019). The oil, as well as the main compounds, have low toxicity upon oral and dermal exposure in experimental animals and showed antioxidant and anti-inflammatory effects, reflecting its protective activity in a cerebral ischemia mouse model (Younis and Mohamed, 2020).

This study was designed to evaluate the larvicidal efficacy of *Santalum album* oil and its nano-emulsion against *Culex pipiens*, and *Aedes aegypti* 3rd instar larvae, and determine their effect on the detoxifying enzymes activity in larval tissues. Oil constituents' determination via gas chromatography-mass spectrometry analysis and oil phenolic compounds determination via liquid chromatography coupled with electrospray ionization and tandem mass spectroscopy.

2. Materials and methods

2.1. Chemicals

Tween 20, sodium glycocholate and sodium cholate hydrate were obtained from Alfa Aesar, Germany. Sandalwood oil purchased from the local market in jazan. Gallic acid, 3.4-dihydroxybenzoic acid, chlorogenic acid, catechin, caffeic acid, methyl gallate, syringic acid, coumaric acid, vanillin, rutin, ellagic acid, ferulic acid, myricetin, daidzein, luteolin, quercetin, naringenin, apegenin, kaempferol and hesperetin as phenolic compounds standards were purchased from Sigma Aldrich, USA. All reagents were HPLC grade.

2.2. Oil nano-emulsion preparation

The oil in water nano-emulsion was prepared by mixing 5 ml of sandalwood oil (at 45°C) in 50 ml beaker contained 10 ml distilled water, 0.5 g sodium cholate, 0.5 g sodium glycocholate and 3.5 ml tween 20 (at 45°C), stirred with magnetic stirrer until a clear emulsion was obtained. The mixture was quenched gradually with 50% v/v water, then, emulsified via sonication for 10 min at 200 W. The nano-emulsion was subjected to freeze drying lyophilization using SP Virtis Advantage Pro Laboratory Benchtop Freeze-Dryer Lyophilizer, with sucrose as a cryoprotectant (Yuan et al., 2008; Gundewadi et al., 2018).

2.3. Characterization of the oil nano-emulsion

2.3.1. Particle size and surface charge using DLS and TEM analysis

The hydrodynamic radius and surface charge were investigated via dynamic light scattering (DLS) to determine the particle size (mean diameter) and zeta potential to confirm stability and uniformity by polydispersity index (PDI) and surface charge using zeta sizer nano Zs analyzer Malvern Panalytical, UK. One mg Sample was dispersed in 10 ml deionized water (Yuan et al., 2008). Particle morphology examined using TEM (Joel-1400 Flash) on carbon coated copper grids (600 mesh). Images were captured using CCD camera (EMT), the accelerating voltage was 80kV (Yuan et al., 2008; Gundewadi et al., 2018).

2.3.2. Scanning Electron microscope (SEM)

Lyophilized sandalwood nano-emulsion sample used to obtain surface images via SEM (Quanta FDG 250, FEL, Hillsboro, OR, USA). The accelerating voltage 20 kV with 10.1 mm working distance (Dubes et al., 2003).

2.3.3. Differential Scanning calorimetry (DSC)

Lyophilized sandalwood nano-emulsion (5 g) was used to investigate the thermal stability profile (DSC-60, Shimadzu, Japan). The sample placed in standard aluminum pans with temperature raized from 2-200 °C covering the thermogram at 10 °C/min (Ji et al., 216).

2.4. Gas chromatography-mass spectrometry (GC-MS)

Chemical composition of oil was determined using gas chromatography-mass spectrometry as detailed previously by El-Kasem Bosly, (2022).

2.5. Liquid chromatography coupled with electrospray ionization and tandem mass spectroscopy (LC-ESI-MS/MS)

Phenolic compounds in the sandalwood oil sample were performed using LC-ESI-MS/MS for the separation. An ExionLC AC HPLC system and SCIEX Triple Quad 5500+MS/MS system equipped with an electrospray ionization for detection. The column, ZORBAX SB-C18 (4.6×100 mm, 1.8 μm) was used. Two mobile phases, A: 0.1 percent formic acid in water and B: acetonitrile in programming mode as follows: 2 percent B from 0 to 1 minute, 2 to 60 percent B from 1 to 21 minutes, 60 percent B from 21 to 25 minutes and 2 percent B from 25.01 to 28 minutes with 0.8 ml/min, as flow rate and the sample was 3 μl in volume. Positive and negative ionization modes were used in the same run in sittings for the multiple reactions monitoring (MRM) of the selected polyphenols as: curtain gas was 25 psi; for sitting positive and negative modes the IonSpray voltage were 4500 and-4500, respectively; source temperature was 400°C; ion source gas 1 and 2 were 55 psi with a declustering potential at 50 V; collision energy at 25 eV and collision energy spread was 10 V.

2.6. *Culex pipiens* and *Aedes aegypti* mosquito colonies

Mosquito larvae of *Culex pipiens* and *Aedes aegypti* were reared as detailed by El-Kasem Bosly, (2022).

2.7. Larvicidal assay

Larvicidal activities of sandalwood essential oil and its nano-emulsion were conducted against *Culex pipiens* and *Aedes aegypti* 3rd instar larvae according to WHO, (2005). Two milliliters of the oil was placed in 100 ml water containing 2% tween 20 and subjected magnetic stirring (CR302, UK). Also, 2 ml of the prepared nano-emulsion was ultrasonicated in 100 ml water for equal distribution. Concentrations were prepared from the aforementioned preparations at 62.5, 125, 250, 500, 1000 and 1500 ppm. Twenty-five larvae from *Cx pipiens* and/or *Ae aegypti* were subjected to every one concentration in glass beakers (250 ml in volume) comprising 150 ml of dechlorinated water (aqueous suspension) at 27± 2 °C, 70± 10% relative humidity and a 12:12 h light/dark photoperiod. The experiment was replicated five times for each concentration per extract and control group (solvent only treated). Larval mortalities were recorded after 24 and 48h.

2.8. Larval preparation for biochemical assays

Third instar larvae of both species were exposed to oil and/or its nano-emulsion at the calculated LD_{50} in three replicates, as well as the control group, according to the aforementioned conditions in the larvicidal assay. Larvae were collected and weighed after 48 h from each group and pooled from each replicate for body homogenization in distilled water 10% (w/v) under ice and via cooling centrifugation at 4 ℃ for 15 min at 10000 rpm the supernatant was used for the biochemical assays.

2.9. Biochemical assays

Larvae supernatants were used for determination of total protein content (Bradford, 1976) and enzyme activities of alkaline phosphatase (Powell and Smith, 1954), α- and β-esterases (Van Asperen, 1962) and glutathione S-transferase (GST) (Habig et al, 1974).

2.10. Data analysis

Percentage larval mortality was calculated according to Abbott, (1925). The larval control mortality was less than 5%, did not need correction according to the WHO, (2005) guidelines. Mortality and biochemical data resulting from all replicates were analyzed by one-way analysis of variance (ANOVA) to find the differences among the activity between each oil or nano-emulsion concentrations using the least significant difference test. Also, all replicates data were subjected to analysis for determination of the larval LC_{50}, LC_{90}, and LC_{95} as well as chi-square values within confidence limits at 95% by using probit analysis and regression between logarithm bas 10 of oil concentration and probit values. Data analysis was done via IBM SPSS Statistics v22 – 64 bit software with statistical significance at $p < 0.05$.

3. Results

3.1. Characteristics of oil nano-emulsion

Sandalwood oil nano-emulsion was characterized via DLS, which revealed particle average size of 195.7 nm and PDI of 0.342 (Figure 1.A), confirming homogeneous, stable and uniform narrow distributed nanoparticles. Zeta potential was -20.1 mV (Figure 1.B). The nano-emulsion morphology detected using TEM is represented in Figure 2(A, B and C), showing regular spherical particles with a size in the range of 112 nm to 196 nm. Scanning electron micrograph (SEM) of lyophilized sandalwood nano-emulsion using sucrose as cryo-protectant was predicted smooth spherical particle shape (Figure 3). Meanwhile, DSC thermogram showed an endothermic melting peak at 181^{0}C (Figure 4).

3.2. Constituents of sandalwood oil by chromatographic analysis.

Gas chromatography-mass spectrometry analysis identified 27 compounds, sesquiterpene alcohols mainly santalols, terpenoids, aromatic compounds, and fatty acid methyl esters (Table 1

and Figure 5). While phenolic compounds that were identified and quantified using LC-ESI-MS/MS analysis were chlorogenic acid (1.15426 ng/ml), ellagic acid (0.14722 ng/ml), luteolin (0.39093 ng/ml), and the highest content was recorded for naringenin (38.08662 ng/ml) (Table 2).

3.3. Larvicidal activities

Larval mortality data are represented in Table 3. In sandalwood oil -exposed groups, after 24 h, *Cx. pipiens* and *Ae aegypti* larvae exhibited 100% mortality at a dose of 1500 ppm with LD_{50}, LD_{90} and LD_{95} identified as 299.47, 847.81, and 1138.73 ppm for *Cx. pipiens* and 349.59, 1011.54, and 1367.06 ppm for *Ae aegypti*, respectively. After 48 h, 100% mortality observed at 1000 and 1500 ppm for *Cx. pipiens* and at 1500 ppm for *Ae aegypti* with LD_{50}, LD_{90}, and LD_{95} identified as 213.01, 617.64, and 835.22 ppm for *Cx. pipiens,* and 250.64, 709.06, and 952.17 ppm for *Ae aegypti*, respectively.

In nano-emulsion-exposed groups, after 24 h, *Cx. pipiens* and *Ae aegypti* larvae exhibited 100% mortality at 1000 ppm, with LD_{50}, LD_{90}, and LD_{95} identified as 187.23, 532.08, and 715.42 ppm for *Cx. pipiens,* and 232.18, 638.98, and 851.39 ppm for *Ae aegypti*, respectively. After 48 h 100% larval mortality was observed at 1000 and 1500 ppm with LD_{50}, LD_{90}, and LD_{95} identified as 137.44, 355.91, and 466.10 ppm for *Cx. pipiens* and 182.37, 468.47, and 630.18 ppm for *Ae aegypti*, respectively.

3.4. Biochemical results

Biochemical results represented in Table 4, showing significant decrease in TP content and ALP and β esterase enzymes activities in *Cx. pipiens* and *Ae. aegypti* exposed to both treatments with significant lowering effect of nano-emulsion as compared to oil. Meanwhile, α esterase and GST enzymes activities showed significant increase upon both treatments as compared to corresponding controls. In addition, nano-emulsion exposed groups showed significant increase in

α esterase and GST enzymes activities in *Cx. pipiens* and *Ae. aegypti* groups comparing to the parallel values in oil exposed groups.

4. Discussion

In recent years, there has been a great interest from health authorities and organizations in the significance of vector-borne diseases at the global and regional levels since they continue to demonstrate a significant health threatening to the societies worldwide (WHO, 2017; Valenzuela and Aksoy, 2018). Mosquito-borne diseases represent the largest measure of this fear, that's because of the mosquitoes ability to transmit many medical and veterinary diseases, like, filariasis, malaria, dengue fever, Rift Valley fever, Lumpy skin, and others which negatively affects human health and causes clear economic losses (Al-Seghayer et al., 1999; Singh et al., 2019). In addition to this interest, research on mosquito control based natural alternative agents instead of synthetic pesticides, with a clear appreciation in the scientific and medical community, especially natural products derived from plants (Şengül Demirak and Canpolat, 2022). Because of their capability to win the goal for reducing pests without harming the environment, essential oils within their chemical constituents, exerted beneficial effects and due to their lipophilic nature acquired the capability for crossing membranes and hence, exerts their toxicity activity towards insects, as well as their antimicrobial, antibacterial, antifungal, antiviral in line with their miscellaneous activities (Stephane and Jules, 2020).

The sandalwood oil nano-emulsion prepared in the present study characterized by Zeta potential was within range -30-30 mV associated with stable nano-emulsion systems and the negative value is necessary for droplet-droplet repulsion and enhanced nano-emulsion stability. In addition, the recorded small PDI that described the degree of particles distribution uniformity in the emulsion confirmed good homogeneity indication (Danaei et al., 2018; Gul et al., 2022). TEM

findings agree with DLS data, however, the particle size determined using TEM was smaller than that detected using DLS due to the sensitivity of technique (Klang et al., 2012). The characteristics of the nano-emulsion were in agreement with previous studies (González et al., 2016; Firooziyan et al., 2021; Zamaniahari et al., 2022). Differential scanning calorimetry can be applied for the recognition of microsponges when loaded molecules are entrapped nearby. Melting, boiling, and/or sublimation points of the entrapped molecules generally change or disappear. According to this, the presented melting peak was thought to be the effect of the sucrose cryo-protectant and no significant endothermic peak was observed for the sandalwood oil nano-emulsion that it was liquid at room temperature.

Both GC-MS and LC-ESI-MS/MS analysis of sandalwood essential oil confirmed the constituents previously recorded (Butaud et al., 2006; Mirsa and Dey, 2012; Bisht et al., 2019; Kucharska et al., 2021; Tripathi et al., 2022).

The larval mortality results confirmed the sandalwood oil effect previously identified against *Ae. aegypti* larvae (Amer and Mehlhorn, 2006). In addition to the efficient larvicidal predicted action against *Cx pipiens, Ae aegypti* and *Aedes albopictus* larvae that reportedly due to the toxicity of the oil constituents (Zhu et al., 2008). Another study showed significant repellant and insecticidal activities of sandalwood oil and its main active ingredients α- and β- santalols against *Aphis gossypii* and suggested sandalwood oil and its main compounds for use as possible ecofriendly management against *Aphis gossypii* (Roh et al., 2015). Sandalwood oil showed a repellent activity for the parasitic mite, *Varroa jacobsoni* which invades and threatens honeybee colonies (Imdorf et al., 1999) and against *Lycoriella mali*, Sciarid flies, with modest activity reported (Choi et al., 2006). Besides, santalol showed activity against the spider mite *Tetranychus urticae* (Roh et al., 2012) acting as acaricidal and oviposition deterring. Furthermore, Indian

sandalwood tree (*S. Album* L.) has benificial properties in inhibiting insects' growth due to its chemical properties (Shankaranarayana et al. 1980).

The study results, showed a significant larvicidal efficacy of the nano-emulsion as compared to that of the oil against both larvae, revealing the enhanced activity of the nano-emulsion in agreement with Duarte et al. (2015), who evaluated rosemary essential oil nano-emulsion and its potential larvicidal effect against *Ae. aegypti* larvae. Moreover, Mahran, (2022) evidenced the larvicidal improvement of basil and cumin essential oils in their nano-emulsion formulations against *Cx. pipiens* larvae.

The sandalwood oil nanoemulsion also recorded significant decrement in total protein contents in the exposed species as compared to their concentrations in the oil exposed larvae and both treatments showed total protein significant decrement as compared to control value, which proposed for the synthesis microsomal detoxifying enzymes (Massoud et al., 2001). The total protein decrement was confirmed in previous studies (Koodalingam et al., 2012; Sugumar et al, 2014). Esterases and GST function as detoxification enzymes for endogenous and exogenous chemicals to eliminate or transform them to less toxic metabolites through different metabolic pathways. The alteration of enzymes throughout the oil compounds action besides the role of enzymes in metabolizing oil constituents was previously proposed (Intirach et al., 2019). Sandalwood oil larvicidal activity was proposed through its target for the detoxifying enzymes (Tong and Bloomquist, 2013) which increased larval sensitivity to tannins and generally for phenolic compounds, in accordance with the predicted sandalwood oil compounds with proposed mosquitocidal activity (Rey et al., 1999; Rey et al., 2001).

Sandalwood oil showed antiviral activity against herpes simplex virus type 1 (HSV-1) in a dose dependent manner and the activity was proposed via oil increment effect on cellular GST

enzyme activity (Benencia and Courrèges, 1999). Noting that β-esterase activity decreased in the present results, which is often the reverse of α-esterase activity as a saver for the larvae from the oil constituents' toxicity. Also, could be because esterase proteins have different substrate specificities resulting in different active sites of the two esterases (Montella et al., 2012). The present results may support the involvement of that enzymes in the detoxification of sandalwood oil or its nanoemulsion in the tested larvae.

Essential oil nano-emulsion protects the oil against oxidation and controls its release and bioactivity by increasing the exposed area and providing the interaction of oil active compounds with their target, resulting in increased stability and shelf life, decreasing degradation due to environmental factors. These properties indicate their effectiveness compared to crude and even pure oil (da Silva et al., 2022). In previous study neem oil nano-emulsion showed effective larvicidal potency aginst *Cx. quinquefasciatus* 3rd instar larvae (Anjali et al., 2012). Balasubramani et al, (2017) showed the larvicidal activity advantage of *Vitex negundo* L. leaf essential oil nano-emulsion (particle size, 200 nm) against *Ae. aegypti* larvae as compared to that of the oil after 12 and 24h. Firooziyan et al, (2021) reported increased larvicidal efficacy of *Cinnamim zelanicum* nano-emulsion against *An. stephensi* larvae compared to the essential oil. Similarly, *Aeollanthus suaveolens* Mart. leaves oil in the nano-emulsion formulation (particle size 126.73 nm and zeta potential -16.25 mV) evaluated larval toxicity *Ae. aegypti* larvae (Lopes Martins et al., 2021).

5. Conclusion

The study verified the enhanced larvicidal potential of sandalwood oil nano-emulsion against *Cx. pipiens* and *Ae. aegypti* mosquito larvae as compared to that of the oil as well as alterations in the detoxifying enzymes based on oil active ingredients. Although the rational use of sandalwood oil is limited as insecticide due to the coast, it is used in a wide range of applications in fragrance and medicinal usage. The insecticidal activity offers a variety of use as a pesticide and the nano-

emulsion formulation adds extra stability and elevates its toxicity against the tested mosquito larvae. The study recommends sandalwood oil nano-emulsion as a safe and stable larvicide against *Cx. pipiens* and *Ae. aegypti* and more biochemical investigations are warranted to explore more larvicidal mode of action.

Acknowledgement: The author wish to acknowledge the Center for Environmental Research and Studies at Jazan University for technical services.

Conflict of interest: The author had no competing interest

Funding: None.

Table ligands and Figures captions

Table 1. Chemical constituents of sandalwood essential oil by gas chromatography-mass spectrometer (GC-MS).

Table 2. Phenolic compound standards versus a sample of sandalwood oil and their quantification (ng/ml) via LC-MS/MS.

Table 3. The larvicidal activities of sandalwood oil and sandalwood nano-emulsion against *Culex pipiens* and *Aedes aegypti* 3rd instar larvae 24 and 48 h post-exposure.

Table 4. Effect sandalwood oil and sandalwood nano-emulsion at LD_{50} on *Culex pipiens* and *Aedes aegypti* 3rd instar larvae

Figure 1. Dynamic light scattering and particle size analysis average particle size of 195.7 nm with PDI of 0.342 **(A)**. Zeta potential and surface charge analysis (-20.1 mV) **(B)**.

Figure 2. Transmission electron microscopy (TEM) of sandalwood oil nano-emulsion. Particle size **(A)** 144–169 nm, **(B)** 117, 137, and 166 nm, **(C)** 112–137 nm.

Figure 3. Scanning electron microscopy (SEM) of sandalwood oil nanoparticles (2000×).

Figure 4. Differential scanning calorimetry of sandalwood oil nanoparticles.

Figure 5. The total ion current chromatograms of sandalwood essential oil chemical constituents detected via GC-MS.

References

Abbott, W.S., 1925. A method of computing the effectiveness of an insecticide. J. Econ. Entomol. 18, 265–267. https://doi.org/10.1093/jee/18.2.265a

Alahmed, A.M., Munawar, K., Khalil, S.M.S., Harbach, R.E., 2019. Assessment and an updated list of the mosquitoes of Saudi Arabia. Parasit. Vectors 12, 356. https://doi.org/10.1186/s13071-019-3579-4

Al-Massarani, S., El-Shaibany, A., Tabanca, N., Ali, A., Estep, A.S., Becnel, J.J., Goger, F., Demirci, B., El-Gamal, A., Baser, K.H.C., 2019. Assessment of selected Saudi and Yemeni plants for mosquitocidal activities against the yellow fever mosquito Aedes aegypti. Saudi Pharm. J. 27, 930–938. https://doi.org/10.1016/j.jsps.2019.07.001

Al-Sarar, A.S., 2010. Insecticide resistance of Culex pipiens (L.) populations (Diptera: Culicidae) from Riyadh city, Saudi Arabia: Status and overcome. Saudi J. Biol. Sci. 17, 95–100. https://doi.org/10.1016/j.sjbs.2010.02.001

Al-Seghayer, S.M., Kenawy, M.A., Ali, O.T.E., 1999. Malaria in the Kingdom of Saudi Arabia: Epidemiology and control. Sci J King Faisal Univ. 1, 6–20.

Amer, A., Mehlhorn, H., 2006. Larvicidal effects of various essential oils against Aedes, Anopheles, and Culex larvae (Diptera, Culicidae). Parasitol. Res. 99, 466–472. https://doi.org/10.1007/s00436-006-0182-3

Anjali, C.H., Sharma, Y., Mukherjee, A., Chandrasekaran, N., 2012. Neem oil (Azadirachta indica) nanoemulsion—a potent larvicidal agent against Culex quinquefasciatus. Pest Manag. Sci. 68, 158–163. https://doi.org/10.1002/ps.2233

Balasubramani, S., Rajendhiran, T., Moola, A.K., Diana, R.K.B., 2017. Development of nanoemulsion from Vitex negundo L. essential oil and their efficacy of antioxidant, antimicrobial and larvicidal activities (Aedes aegypti L.). Environ. Sci. Pollut. Res. 24, 15125–15133. https://doi.org/10.1007/s11356-017-9118-y

Benencia, F., Courrèges, M.C., 1999. Antiviral activity of sandalwood oil against Herpes simplex viruses-1 and -2. Phytomedicine 6, 119–123. https://doi.org/https://doi.org/10.1016/S0944-7113(99)80046-4

Bisht, S.S., Ravindra, M., Gayathri, D.N. (2019). Variability in yield and composition of oil from Indian Sandalwood (Santalum album L.) trees grown in homogeneous conditions. Tropical Plant Research. 6(1), 31–36. https://doi.org/10.22271/tpr.2019.v6.i1.006

Bosly, H. A., 2015. Larvicidal activity of Thymus Vulgaris Essential oil against Aedes Aegypti L.(Diptera: Culicidae). Indian J. Entomol. 77, 405–409. https://doi.org/10.5958/0974-8172.2015.00077.2

Bradford, M.M., 1976. A rapid and sensitive method for the quantitation of microgram quantities of protein utilizing the principle of protein-dye binding. Anal. Biochem. 72, 248–254. https://doi.org/10.1016/0003-2697(76)90527-3

Butaud, J.-F., Raharivelomanana, P., Bianchini, J.-P., Faure, R., Gaydou, E.M., 2006. Leaf C-glycosylflavones from Santalum insulare (Santalaceae). Biochem. Syst. Ecol. 34, 433–435. https://doi.org/10.1016/j.bse.2005.11.012

Choi, W.-S., Park, B.-S., Lee, Y.-H., Yoon, H.Y., Lee, S.-E., 2006. Fumigant toxicities of essential oils and monoterpenes against Lycoriella mali adults. Crop Prot. 25, 398–401. https://doi.org/10.1016/j.cropro.2005.05.009

da Silva, B.D., do Rosário, D.K.A., Weitz, D.A., Conte-Junior, C.A., 2022. Essential oil nanoemulsions: Properties, development, and application in meat and meat products. Trends Food Sci. Technol.121, 1-13. https://doi.org/10.1016/j.tifs.2022.01.026

Danaei, M., Dehghankhold, M., Ataei, S., Hasanzadeh Davarani, F., Javanmard, R., Dokhani, A., Khorasani, S., Mozafari, M. R., 2018. Impact of Particle Size and Polydispersity Index on the Clinical Applications of Lipidic Nanocarrier Systems. Pharmaceutics, 10, 57. https://doi.org/10.3390/pharmaceutics10020057

Duarte, J.L., Amado, J.R.R., Oliveira, A.E., Cruz, R.A.S., Ferreira, A.M., Souto, R.N.P., Falcão, D.Q., Carvalho, J.C.T., Fernandes, C.P., 2015. Evaluation of larvicidal activity of a nanoemulsion of Rosmarinus officinalis essential oil. Rev. Bras. Farmacogn. 25, 189–192. https://doi.org/10.1016/j.bjp.2015.02.010

Dubes, A., Parrot-Lopez, H., Abdelwahed, W., Degobert, G., Fessi, H., Shahgaldian, P., Coleman, A.W., 2003. Scanning electron microscopy and atomic force microscopy imaging of solid lipid nanoparticles derived from amphiphilic cyclodextrins. Eur. J. Pharm. Biopharm. 55, 279–282. https://doi.org/10.1016/S0939-6411(03)00020-1

El-Kasem Bosly, H.A., 2022. Larvicidal and adulticidal activity of essential oils from plants of the Lamiaceae family against the West Nile virus vector, Culex pipiens (Diptera: Culicidae). Saudi J. Biol. Sci. 29, 103350. https://doi.org/https://doi.org/10.1016/j.sjbs.2022.103350

Endersby-Harshman, N.M., Ali, A., Alhumrani, B., Alkuriji, M.A., Al-Fageeh, M.B., Al-Malik, A., Alsuabeyl, M.S., Elfekih, S., Hoffmann, A.A., 2021. Voltage-sensitive sodium channel

(Vssc) mutations associated with pyrethroid insecticide resistance in Aedes aegypti (L.) from two districts of Jeddah, Kingdom of Saudi Arabia: baseline information for a Wolbachia release program. Parasit. Vectors 14, 361. https://doi.org/10.1186/s13071-021-04867-3

Firooziyan, S., Amani, A., Osanloo, M., Moosa-Kazemi, S.H., Basseri, H.R., Hajipirloo, H.M., Sadaghianifar, A., Sedaghat, M.M., 2021. Preparation of nanoemulsion of Cinnamomum zeylanicum oil and evaluation of its larvicidal activity against a main malaria vector Anopheles stephensi. J. Environ. Heal. Sci. Eng. 19, 1025–1034. https://doi.org/10.1007/s40201-021-00667-0

González, J.W., Yeguerman, C., Marcovecchio, D., Delrieux, C., Ferrero, A., Band, B.F., 2016. Evaluation of sublethal effects of polymer-based essential oils nanoformulation on the german cockroach. Ecotoxicol. Environ. Saf. 130, 11–18. https://doi.org/10.1016/j.ecoenv.2016.03.045

Gul, U., Khan, M.I., Madni, A., Sohail, M.F., Rehman, M., Rasul, A., Peltonen, L., 2022. Olive oil and clove oil-based nanoemulsion for topical delivery of terbinafine hydrochloride: in vitro and ex vivo evaluation. Drug Deliv. 29, 600–612. https://doi.org/10.1080/10717544.2022.2039805

Gundewadi, G., Sarkar, D.J., Rudra, S.G., Singh, D., 2018. Preparation of basil oil nanoemulsion using *Sapindus mukorossi* pericarp extract: Physico-chemical properties and antifungal activity against food spoilage pathogens. Ind. Crops Prod. 125, 95–104. https://doi.org/10.1016/j.indcrop.2018.08.076

Habig, W.H., Pabst, M.J., Jakoby, W.B., 1974. Glutathione S-transferases: the first enzymatic step in mercapturic acid formation. J. Biol. Chem. 249, 7130–7139.

Imdorf, A., Bogdanov, S., Ochoa, R.I., Calderone, N.W., 1999. Use of essential oils for the control of Varroa jacobsoni Oud. in honey bee colonies. Apidologie 30, 209–228. https://doi.org/10.1051/apido:19990210

Intirach, J., Junkum, A., Lumjuan, N., Chaithong, U., Somboon, P., Jitpakdi, A., Riyong, D., Champakaew, D., Muangmoon, R., Chansang, A., 2019. Biochemical effects of Petroselinum crispum (Umbellifereae) essential oil on the pyrethroid resistant strains of Aedes aegypti (Diptera: Culicidae). Insects 10, 1-19. https://doi.org/10.3390/insects10010001

Jaiswal M, Dudhe R, and Sharma PK, Nanoemulsion: an advanced mode of drug delivery system, *3 Biotech* **5**:123–127, Springer (2015). https://doi.org/10.1007/s13205-014-0214-0

Ji, P., Yu, T., Liu, Y., Jiang, J., Xu, J., Zhao, Y., Hao, Y., Qiu, Y., Zhao, W., Wu, C., 2016. Naringenin-loaded solid lipid nanoparticles: preparation, controlled delivery, cellular uptake, and pulmonary pharmacokinetics. Drug Des. Devel. Ther. 10, 911-925. https://doi.org/10.2147/DDDT.S97738

Klang, V., Matsko, N.B., Valenta, C., Hofer, F., 2012. Electron microscopy of nanoemulsions: An essential tool for characterisation and stability assessment. Micron 43, 85–103. https://doi.org/https://doi.org/10.1016/j.micron.2011.07.014

Koodalingam, A., Mullainadhan, P., Rajalakshmi, A., Deepalakshmi, R., Ammu, M., 2012. Effect of a Bt-based product (Vectobar) on esterases and phosphatases from larvae of the mosquito Aedes aegypti. Pestic. Biochem. Physiol. 104, 267–272. https://doi.org/10.1016/j.pestbp.2012.09.008

Kucharska, M., Frydrych, B., Wesolowski, W., Szymanska, J.A., Kilanowicz, A., 2021. A comparison of the composition of selected commercial sandalwood oils with the International Standard. Molecules 26(8), 2249. https://doi.org/10.3390/molecules26082249

Lopes Martins, R., Bruno Lobato Rodrigues, A., de Menezes Rabelo, É., Lima Santos, L., Barreto Brandão, L., Gomes Faustino, C., Luzia Ferreira Farias, A., Maria da Cunha Sá, D., de Castro Cantuária, P., Kardec Ribeiro Galardo, A., Susan Moreira da Silva de Almeida, S., 2021. Development of larvicide nanoemulsion from the essential oil of Aeollanthus suaveolens Mart. ex Spreng against Aedes aegypti, and its toxicity in non-target organism. Arab. J. Chem. 14, 103148. https://doi.org/https://doi.org/10.1016/j.arabjc.2021.103148

Mahran, H.A., 2022. Using nanoemulsions of the essential oils of a selection of medicinal plants from Jazan, Saudi Arabia, as a green larvicidal against Culex pipiens. PLoS One 17, e0267150. https://doi.org/10.1371/journal.pone.0267150

Massoud, A.M., Labib, I.M., Rady, M., 2001. Biochemical changes of Culex pipiens larvae treated with oil and oleo-resin extracts of Myrrh Commiphora molmol. J. Egypt. Soc. Parasitol. 31, 517—529. PMID: 11478451

McClements, D. J. Nanoemulsions versus microemulsions: Terminology, differences, and similarities. *Soft Matter* **8,** 1719–1729 (2012). https://doi.org/10.1039/c2sm06903b

Misra, B.B., Dey, S., 2012. Differential extraction and GC-MS based quantification of sesquiterpenoids from immature heartwood of East Indian sandalwood tree. J. Nat. Sci. Res. 2, 29–33. http://www.iiste.org/Journals/index.php/JNSR/article/view/2580

Montella, I.R., Schama, R., Valle, D., 2012. The classification of esterases: an important gene family involved in insecticide resistance-A review. Mem. Inst. Oswaldo Cruz 107, 437–449. https://doi.org/10.1590/S0074-02762012000400001

Powell, M.E.A., Smith, M.J.H., 1954. The determination of serum acid and alkaline phosphatase activity with 4-aminoantipyrine (AAP). J. Clin. Pathol. 7, 245–248. https://doi.org/10.1136/jcp.7.3.245

Rey, D., Cuany, A., Pautou, M.-P., Meyran, J.-C., 1999. Differential sensitivity of mosquito taxa to vegetable tannins. J. Chem. Ecol. 25(3), 537–548. https://doi.org/10.1023/A:1020953804114

Rey, D., David, J.P., Besnard, G., Jullien, J.L., Lagneau, C., Meyran, J.C., 2001. Comparative sensitivity of larval mosquitoes to vegetable polyphenols versus conventional insecticides. Entomol. Exp. Appl. 98, 361–367. https://doi.org/10.1046/j.1570-7458.2001.00793.x

Roh, H.S., Kim, J., Shin, E.-S., Lee, D.W., Choo, H.Y., Park, C.G., 2015. Bioactivity of sandalwood oil (Santalum austrocaledonicum) and its main components against the cotton aphid, Aphis gossypii. J. Pest Sci. 88, 621–627. https://doi.org/10.1007/s10340-014-0631-1

Roh, H.S., Park, K.C., Park, C.G., 2012. Repellent effect of santalol from sandalwood oil against Tetranychus urticae (Acari: Tetranychidae). J. Econ. Entomol. 105, 379–385. https://doi.org/10.1603/EC11262

Şengül Demirak, M.Ş., Canpolat, E., 2022. Plant-Based Bioinsecticides for Mosquito Control: Impact on Insecticide Resistance and Disease Transmission. Insects 13, 162. https://doi.org/10.3390/insects13020162

Shankaranarayana, K.H., Ayyar, K.S., Rao, G.S.K., 1980. Insect growth inhibitor from the bark of Santalum album. Phytochemistry 19, 1239–1240. https://doi.org/10.1016/0031-9422(80)83096-2

Singh, H., Singh, O.P., Akhtar, N., Sharma, G., Sindhania, A., Gupta, N., Valecha, N., 2019. First report on the transmission of Zika virus by Aedes (Stegomyia) aegypti (L.)(Diptera: Culicidae) during the 2018 Zika outbreak in India. Acta Trop. 199, 105114. https://doi.org/10.1016/j.actatropica.2019.105114

Stephane, F.F.Y., Jules, B.K.J., 2020. Terpenoids as Important Bioactive Constituents of Essential Oils. In Essential Oils - Bioactive Compounds, New Perspectives and Applications. IntechOpen. https://doi.org/10.5772/intechopen.91426

Sugumar, S., Clarke, S.K., Nirmala, M.J., Tyagi, B.K., Mukherjee, A., Chandrasekaran, N., 2014. Nanoemulsion of eucalyptus oil and its larvicidal activity against Culex quinquefasciatus. Bull. Entomol. Res. 104, 393–402. https://doi.org/10.1017/S0007485313000710

Tong, F., Bloomquist, J.R., 2013. Plant essential oils affect the toxicities of carbaryl and permethrin against Aedes aegypti (Diptera: Culicidae). J. Med. Entomol. 50, 826–832. https://doi.org/10.1603/ME13002

Tripathi, S., Kumar, P., Rout, P.K., Khare, S.K., Naik, S., 2022. Comparison of yield and quality of sandalwood oil extracted from heartwood of trees cultivated in different states of India. Mater. Today Proc. 57, 2400–2405. https://doi.org/10.1016/j.matpr.2021.12.536

Valenzuela, J.G., Aksoy, S., 2018. Impact of vector biology research on old and emerging neglected tropical diseases. PLoS Negl. Trop. Dis. https://doi.org/10.1371/journal.pntd.0006365

Van Asperen, K., 1962. A study of housefly esterases by means of a sensitive colorimetric method. J. Insect Physiol. 8, 401–416. https://doi.org/10.1016/0022-1910(62)90074-4

Wilson, A.L., Courtenay, O., Kelly-Hope, L.A., Scott, T.W., Takken, W., Torr, S.J., Lindsay, S.W., 2020. The importance of vector control for the control and elimination of vector-borne diseases. PLoS Negl. Trop. Dis. 14, e0007831. https://doi.org/10.1371/journal.pntd.0007831

World Health Organization, 2013. Larval source management: a supplementary malaria vector control measure: an operational manual. 1–2. https://apps.who.int/iris/bitstream/handle/10665/85379/9789241505604_eng.pdf

World Health Organization. 2005. Guidelines for laboratory and field testing of mosquito larvicides. World Health Organization, 1–41. Retrieved from http://whqlibdoc.who.int/hq/2005/WHO_CDS_WHOPES_GCDPP_2005.13.pdf?ua=1

World Health Organization. 2017. Global Vector Control Response (Vol. 4, pp. 57–71). Retrieved from http://marefateadyan.nashriyat.ir/node/150

Younis, N.S., Mohamed, M.E., 2020. Sandalwood oil neuroprotective effects on middle cerebral artery occlusion model of ischemic brain stroke. Pharmacogn. Mag. 16, 117-122. https://doi.org/10.4103/pm.pm_398_19

Yuan, Y., Gao, Y., Zhao, J., Mao, L., 2008. Characterization and stability evaluation of β-carotene nanoemulsions prepared by high pressure homogenization under various emulsifying conditions. Food Res. Int. 41, 61–68. https://doi.org/10.1016/j.foodres.2007.09.006

Zamaniahari, S., Jamshidi, A., Moosavy, M.-H., Khatibi, S.A., 2022. Preparation and evaluation of Mentha spicata L. essential oil nanoemulsion: physicochemical properties, antibacterial activity against foodborne pathogens and antioxidant properties. J. Food Meas. Charact. https://doi.org/10.1007/s11694-022-01436-9

Zhang, X., Niu, M., Teixeira da Silva, J.A., Zhang, Y., Yuan, Y., Jia, Y., Xiao, Y., Li, Y., Fang, L., Zeng, S., 2019. Identification and functional characterization of three new terpene synthase genes involved in chemical defense and abiotic stresses in Santalum album. BMC Plant Biol. 19, 1–18. https://doi.org/10.1186/s12870-019-1720-3

Zhu, J., Zeng, X., O'neal, M., Schultz, G., Tucker, B., Coats, J., Bartholomay, L., Xue, R.-D., 2008. Mosquito larvicidal activity of botanical-based mosquito repellents. J. Am. Mosq. Control Assoc. 24, 161–168. https://doi.org/10.2987/8756-971X(2008)24[161:MLAOBM]2.0.CO;2

Evaluation of Larvicidal Enhanced Activity of Sandalwood Oil via Nano-emulsion against Culex Pipiens and Ades Aegypti

ORIGINALITY REPORT

14%
SIMILARITY INDEX

PRIMARY SOURCES

1	Hanan Abo El-Kasem Bosly. "Larvicidal and adulticidal activity of essential oils from plants of the Lamiaceae family against the West Nile virus vector, Culex pipiens (Diptera: Culicidae)", Saudi Journal of Biological Sciences, 2022<br>Crossref	45 words — 1%
2	Ibrahim Taha Radwan, Mohamed M. Baz, Hanem Khater, Abeer Mousa Alkhaibari, Abdelfattah M. Selim. "Mg-LDH Nanoclays Intercalated Fennel and Green Tea Active Ingredient: Field and Laboratory Evaluation of Insecticidal Activities against Culex pipiens and Their Non-Target Organisms", Molecules, 2022<br>Crossref	39 words — 1%
3	www.science.gov<br>Internet	26 words — 1%
4	docksci.com<br>Internet	23 words — 1%
5	bio-protocol.org<br>Internet	17 words — < 1%
6	Hoda S. M. Abdel-Ghany, Sobhy Abdel-Shafy, Mai Abuowarda, Rabab M. El-Khateeb, Essam M. Hoballah, Magdy M. Fahmy. "Acaricidal activity of Artemisia	16 words — < 1%

herba-alba and Melia azedarach oil nanoemulsion against Hyalomma dromedarii and their toxicity on Swiss albino mice", Experimental and Applied Acarology, 2021
Crossref

7 Samira Firooziyan, Mahmoud Osanloo, Hamid Reza Basseri, Seyed Hasan Moosa-Kazemi et al. "Nanoemulsion of Myrtus communis essential oil and evaluation of its larvicidal activity against Anopheles stephensi", Arabian Journal of Chemistry, 2022 — 14 words — < 1%
Crossref

8 biblio.ugent.be — 14 words — < 1%
Internet

9 bmcgenomics.biomedcentral.com — 13 words — < 1%
Internet

10 peerj.com — 13 words — < 1%
Internet

11 www.researchsquare.com — 13 words — < 1%
Internet

12 www.j3.jstage.jst.go.jp — 12 words — < 1%
Internet

13 Asma Fraj, Fadhel Jaâfar, Meritxell Marti, Luisa Coderch, Neji Ladhari. "A comparative study of oregano (Origanum vulgare L.) essential oil-based polycaprolactone nanocapsules/ microspheres: Preparation, physicochemical characterization, and storage stability", Industrial Crops and Products, 2019 — 11 words — < 1%
Crossref

14 John C Boik, Robert A Newman. "A classification model to predict synergism/antagonism of — 11 words — < 1%

cytotoxic mixtures using protein-drug docking scores", BMC Pharmacology, 2008
Crossref

15 Mohamed M. Baz, Abdelfattah Selim, Ibrahim Taha Radwan, Abeer Mousa Alkhaibari, Hanem F. Khater. "Larvicidal and adulticidal effects of some Egyptian oils against Culex pipiens", Scientific Reports, 2022 — 11 words — < 1%
Crossref

16 hal.univ-lorraine.fr — 11 words — < 1%
Internet

17 mdpi-res.com — 11 words — < 1%
Internet

18 www.seekdl.org — 11 words — < 1%
Internet

19 B. Sundararajan, Anil Kumar Moola, K. Vivek, B.D.Ranjitha Kumari. "Formulation of nanoemulsion from leaves essential oil of Ocimum basilicum L. and its antibacterial, antioxidant and larvicidal activities (Culex quinquefasciatus)", Microbial Pathogenesis, 2018 — 10 words — < 1%
Crossref

20 bioone.org — 10 words — < 1%
Internet

21 link.springer.com — 10 words — < 1%
Internet

22 www.aimspress.com — 10 words — < 1%
Internet

23 www.parasite-journal.org — 10 words — < 1%
Internet

24 www.yumpu.com
Internet
10 words — < 1%

25 "Handbook of Ecomaterials", Springer Nature, 2019
Crossref
9 words — < 1%

26 "The Sandalwood Genome", Springer Science and Business Media LLC, 2022
Crossref
9 words — < 1%

27 Alejandro Lucia, Eduardo Guzmán. "Emulsions containing essential oils, their components or volatile semiochemicals as promising tools for insect pest and pathogen management", Advances in Colloid and Interface Science, 2021
Crossref
9 words — < 1%

28 Kamal Gholamipourfard, Mehdi Salehi, Erika Banchio. "Mentha piperita phytochemicals in agriculture, food industry and medicine: Features and applications", South African Journal of Botany, 2021
Crossref
9 words — < 1%

29 Mandeep Kaur, Pooja Chadha, Sanehdeep Kaur, Amarjeet Kaur, Rajvir Kaur. "Schizophyllum commune induced oxidative stress and immunosuppressive activity in Spodoptera litura", Research Square Platform LLC, 2020
Crossref Posted Content
9 words — < 1%

30 Nehad E.M. Taktak, Mohamed E.I. Badawy, Osama M. Awad, Nadia E. Abou El-Ela. "Nanoemulsions containing some plant essential oils as promising formulations against Culex pipiens (L.) larvae and their biochemical studies", Pesticide Biochemistry and Physiology, 2022
Crossref
9 words — < 1%

31	Raymond Poon, Hugh Davis, Pierre Le. "EFFECTS OF BENZOTHIOPHENE ON MALE RATS FOLLOWING SHORT-TERM ORAL EXPOSURE", Journal of Toxicology and Environmental Health, 1997<br>Crossref	9 words — < 1%
32	T. Sarita Achari, Chinmayee Panda, Tapan Kumar Barik. "Biochemical response of Aedes aegypti and Aedes albopictus mosquitoes after exposure to thermal stress and toxin of Bacillus thuringiensis israelensis", International Journal of Tropical Insect Science, 2021<br>Crossref	9 words — < 1%
33	assets.researchsquare.com<br>Internet	9 words — < 1%
34	beta.space.iknito.com<br>Internet	9 words — < 1%
35	cms.galenos.com.tr<br>Internet	9 words — < 1%
36	ttngmai.files.wordpress.com<br>Internet	9 words — < 1%
37	www.ncbi.nlm.nih.gov<br>Internet	9 words — < 1%
38	Erdal Dinç, Abdil Ozdemir, Halil Aksoy, Dumitru Baleanu. "Chemometric Approach to Simultaneous Chromatographic Determination of Paracetamol and Chlorzoxazone in Tablets and Spiked Human Plasma", Journal of Liquid Chromatography & Related Technologies, 2007<br>Crossref	8 words — < 1%
39	Jean-Philippe David. "Larvicidal properties of decomposed leaf litter in the subalpine mosquito	8 words — < 1%

breeding sites", Environmental Toxicology and Chemistry, 01/2002
Crossref

40 K. V. Sumitha, John E. Thoppil. "Larvicidal efficacy and chemical constituents of O. gratissimum L. (Lamiaceae) essential oil against Aedes albopictus Skuse (Diptera: Culicidae)", Parasitology Research, 2015
Crossref
8 words — < 1%

41 Kesavan Subaharan, Periyasamy Senthamarai Selvan, Thagare Manjunatha Subramanya, Rajendran Senthoorraja et al. "Ultrasound-assisted nanoemulsion of Trachyspermum ammi essential oil and its constituent thymol on toxicity and biochemical aspect of Aedes aegypti", Environmental Science and Pollution Research, 2022
Crossref
8 words — < 1%

42 P. Mishra, A.P.B Balaji, P.K. Dhal, R.S. Suresh Kumar, S. Magdassi, K. Margulis, B.K Tyagi, A. Mukherjee, N. Chandrasekaran. "Stability of nano-sized permethrin in its colloidal state and its effect on the physiological and biochemical profile of Culex tritaeniorhynchus larvae", Bulletin of Entomological Research, 2017
Crossref
8 words — < 1%

43 discovery.researcher.life
Internet
8 words — < 1%

44 eprints.uthm.edu.my
Internet
8 words — < 1%

45 google.com.na
Internet
8 words — < 1%

46 www.bu.edu.eg
Internet
8 words — < 1%

47	www.mdpi.com<br>Internet	8 words — < 1%
48	"Mosquito-borne Diseases", Springer Science and Business Media LLC, 2018<br>Crossref	7 words — < 1%
49	Jin-Jun Wang, Zhi-Mo Zhao, James H Tsai. "Resistance and some enzyme activities in Liposcelis bostrychophila Badonnel (Psocoptera: Liposcelididae) in relation to carbon dioxide enriched atmospheres", Journal of Stored Products Research, 2000<br>Crossref	6 words — < 1%
50	Mojgan Heydari, Amirmostafa Amirjani, Mozhgan Bagheri, Iman Sharifian, Qodrat Sabahi. "Eco-friendly pesticide based on peppermint oil nanoemulsion: preparation, physicochemical properties, and its aphicidal activity against cotton aphid", Environmental Science and Pollution Research, 2019<br>Crossref	6 words — < 1%
51	Sayed Mohammad Sahafi, Sayed Amir Hossein Goli, Mahdi Kadivar, Jaleh Varshosaz. "Preparation and characterization of bioactive oils nanoemulsions: Effect of oil unsaturation degree, emulsifier type and concentration", Journal of Dispersion Science and Technology, 2017<br>Crossref	6 words — < 1%

EXCLUDE QUOTES	ON	EXCLUDE SOURCES	OFF
EXCLUDE BIBLIOGRAPHY	ON	EXCLUDE MATCHES	OFF
